# Derivation and internal–external validation of clinical prediction model for postoperative clinically important hypotension in patients undergoing noncardiac surgery: an international prospective cohort study^☆^

**DOI:** 10.1016/j.bjao.2025.100410

**Published:** 2025-05-22

**Authors:** Stephen Su Yang, German Malaga, Maria Lazo-Porras, Patricia Busta-Flores, Aida del Carmen Rotta-Rotta, Pavel S. Roshanov, Daniel I. Sessler, Amal Bessissow, Thomas Schricker, Vicky Tagalakis, Diane Heels-Ansdell, Shirley Pettit, P.J. Devereaux

**Affiliations:** 1 Department of Anesthesia, Jewish General Hospital, McGill University, Montreal, QC, Canada; 2 Lady Davis Research Institute, Jewish General Hospital, McGill University, Montreal, QC, Canada; 3 CONEVID Unidad de Conocimiento y Evidencia, Facultad de Medicina, Universidad Peruana Cayetano Heredia, Lima, Peru; 4 Hospital Nacional Cayetano Heredia, Lima, Peru; 5 Division of Nephrology, Department of Medicine, Western University, London, ON, Canada; 6 Department of Anesthesiology, University of Texas Health Science Center, Houston, TX, USA; 7 Department of Medicine, McGill University Health Centre, McGill University, Montreal, QC, Canada; 8 Department of Anesthesia, McGill University Health Centre, McGill University, Montreal, QC, Canada; 9 Division of General Internal Medicine, Jewish General Hospital, Montreal, QC, Canada; 10 Centre for Clinical Epidemiology of the Lady Davis Institute for Medical Research, Jewish General Hospital, Montreal, QC, Canada; 11 Department of Health Research Methods, Evidence, and Impact, McMaster University, Hamilton, ON, Canada; 12 Population Health Research Institute, Hamilton, ON, Canada; 13 Department of Medicine, McMaster University, Hamilton, ON, Canada

**Keywords:** hypotension, noncardiac, perioperative, prediction model, surgical specialities

## Abstract

**Background:**

Intraoperative and postoperative hypotension are associated with myocardial injury/infarction, stroke, acute kidney injury, and death. Because of its prolonged duration, postoperative hypotension contributes more to the risk of organ injury compared with intraoperative hypotension. A prediction model for clinically important postoperative hypotension after noncardiac surgery is needed to guide clinicians.

**Methods:**

We performed a secondary analysis of the Vascular Events in Noncardiac Surgery Patients Cohort Evaluation (VISION) study. Patients aged ≥45 yr who had inpatient noncardiac surgery across 28 centres in 14 countries were included. In 14 of the centres selected at random (derivation cohort), we evaluated 49 variables using logistic regression to develop a model to predict postoperative clinically important hypotension, defined as a systolic blood pressure ≤90 mm Hg, that resulted in clinical intervention. The postoperative period was defined from the Post-Anesthesia Care Unit to hospital discharge. We then evaluated its calibration and discrimination in the other 14 centres (validation cohort).

**Results:**

Among 40 004 patients in VISION, 20 442 (51.1%) were included in the derivation cohort, and 19 562 (48.9%) patients were included in the validation cohort. The incidence of clinically important postoperative hypotension in the entire cohort was 12.4% (4959 patients). A 41-variable model predicted the risk of clinically important postoperative hypotension (bias-corrected C-statistic: 0.73, C-statistic in validation cohort: 0.72). A simplified prediction model also predicted clinically important hypotension (bias-corrected C-statistic: 0.68) based on four information items.

**Conclusions:**

Postoperative clinically important hypotension may be estimated before surgery using our primary model and a simple four-element model.

**Clinical trial registration:**

NCT00512109.

Worldwide, more than 230 million noncardiac surgeries are performed annually.^1^ Despite advances in surgical technique and perioperative management, postoperative medical complications remain common. In surgical patients aged ≥45 yr, >10% will suffer a major cardiovascular event within 30 days, including mortality, myocardial injury after noncardiac surgery (MINS), or stroke.^2^ These complications are often preceded by a period of hypotension.^3^

Perioperative hypotension is common; however, multiple definitions of perioperative hypotension exist, using systolic or mean arterial blood pressure, and the threshold varies between absolute numbers or relative numbers.^4^ The definition of clinically important hypotension, defined as systolic blood pressure (SBP) <90 mm Hg that required a clinical intervention, was strongly associated with major postoperative cardiovascular complications.^5^ In a large international RCT, Perioperative Ischemic Evaluation-1 (POISE-1) trial, of 8351 patients from 190 centres in 23 countries, clinically important hypotension had the highest population attributable risk (PAR) for death compared with all other risk factors (PAR 37.3%) and stroke (PAR 14.7%).^3^ A similar association is seen between clinically important hypotension and perioperative myocardial infarction (MI). In POISE-2, a factorial RCT of 10 010 patients from 135 centres in 23 countries, clinically important hypotension that preceded the event was found to be an independent predictor of perioperative MI (adjusted hazard ratio [aHR] 1.37; 95% confidence interval [CI] 1.16–1.62).^6^ Available evidence thus indicates that clinically important hypotension is strongly associated with postoperative cardiovascular complications and mortality.

Although intraoperative hypotension is common, an appreciable proportion of patients experience postoperative hypotension. In an international multicentred cohort study (*n*=14 687), 19.5% of patients experienced at least one episode of postoperative hypotension.^7^ The most striking difference between intraoperative and postoperative periods was the duration of hypotension. The median duration of intraoperative hypotension was 15 min, 30 min in the Post-Anesthesia Care Unit (PACU), and 150 min on postoperative day 1.^8^ Patients who experienced an episode of postoperative hypotension were at twice the risk of postoperative major cardiovascular events (i.e. death, MINS, or stroke) (adjusted odds ratio [aOR] 2.14; 95% CI 1.89–2.43).^7^^,^^9^ Our goal was thus to create clinical prediction models to provide an estimation of the expected risk for clinically important postoperative hypotension (CIPH).

## Methods

### Study design

We conducted the Vascular Events in Noncardiac Surgery Patient Cohort Evaluation (VISION) prospective international cohort study that included 40 004 patients from 28 centres in 14 countries throughout North and South America, Africa, Asia, Australia, and Europe. Patients were recruited from August 2007 to November 2013 (NCT00512109). Research ethics board or institutional review board approval was obtained at each participating site. Patients provided written informed consent before surgery, and for those from whom we could not obtain preoperative consent (e.g. emergency surgery) research staff obtained consent within 24 h after surgery. We previously reported details of the study design and methods.^10^ Surgical patients who were ≥45 yr old and underwent an inpatient noncardiac surgery (i.e. who required an overnight stay in hospital after surgery) that required general or regional anaesthesia were included in the study. Central data consistency checks and on-site monitoring were used as part of the data quality process. The Strengthening of the Reporting of Observational Studies in Epidemiology (STROBE) guidelines and the Transparent Reporting of a multivariable Prediction model for Individual prognosis or diagnosis (TRIPOD) guidelines were followed in this report.

### Statistical analyses

STATA MP version 15 (StataCorp LLC, College Station, TX, USA) and R version 3.6.2 (R Foundation for Statistical Computing, Vienna, Austria) were used for all analyses with *-rms* package used in R.

### Data splitting

The entire cohort was divided into two cohorts: one used for model development and the other one used for model validation. The cohort was split at the centre level and randomly selected to be included in either derivation or validation cohort. Each centre in the derivation cohort was matched to another centre in the validation cohort with a similar geographic location (i.e. country or region), similar profile, or both of surgical procedure.

### Descriptive statistics

Patient characteristics were summarised as follows. Continuous variables were reported using mean and standard deviation (sd) or median and inter-quartile range (IQR), and independent Student's *t*-tests were used to test for differences between the derivation and validation cohorts. Categorical and binary variables were reported using frequency distribution, and Pearson's χ^2^ tests were used to compare the difference between the derivation and validation cohorts. All tests were two-sided and significance was defined as *P*<0.05.

### Outcome variable

The outcome variable was the presence of CIPH, defined as SBP ≤90 mm Hg, where a clinician intervened to improve the patient's haemodynamics during the postoperative period, which was the period from PACU to hospital discharge. These interventions included crystalloid or colloid administration, blood transfusion, vasopressor, inotropes, or installation of an intra-aortic balloon pump.

### Development and validation of clinical prediction model

A multivariable logistic regression analysis was performed to develop a prediction model in which the dependent variable was the occurrence of CIPH. The independent variables were selected for entry into the model based on biological plausibility and a literature review of known associated factors related to perioperative hypotension. These variables were all predefined and occurred before surgery. A backward elimination approach, planned *a priori*, with a *P*-value >0.10 was used as a criterion for removal.^11^ The type of surgery was forced for entry into the final model. All continuous variables were converted to categorical variables to account for the potential of non-linear relationships and to facilitate clinicians in interpreting the data. The degree of multicollinearity was tested using the variance inflation factor (VIF); if two covariates were highly correlated (i.e. VIF >5), the least significant variable was dropped from the model (Supplementary Table S4).

Model performance was evaluated using an internal–external validation approach and described using calibration and discrimination.^12^^,^^13^ Calibration was assessed graphically by comparing the probabilities of the predicted and observed events. Discrimination was quantified using the concordance statistics (C-statistics). Internal validation was conducted on the derivation cohort using bootstrap resampling and iteration of the entire modelling process, including backward elimination across 1000 samples, to correct for optimism as a result of overfitting.^14^ Both calibration plots and C-statistics reported optimism correction. The model's performance was subsequently examined using cross-validation in the validation cohort, with both calibration and discrimination metrics reported.

### Simplified score system

A simplified risk model scoring system was developed to allow for easier prediction. Four independent variables with the highest χ^2^ from the prediction of CIPH were selected for inclusion in the simplified risk index. Using an internal–external validation process, model calibration and discrimination were evaluated in the derivation cohort using bootstrapping and then the model was evaluated in the validation cohort.

### Approach to missing data

The primary analysis included only patients with complete predictor and outcome data.

### Sensitivity analyses

Three secondary models were developed and evaluated in the same way as the primary model: one to determine if the addition of intraoperative variables could improve model performance, a second to determine if the addition of perioperative antihypertensives improved model performance, and a third to determine if single stochastic conditional imputation (with logistic regression for binary variables and predictive mean matching for continuous variables) affected performance. We performed a sensitivity analysis using the same predictors as the primary model on the outcome of postoperative hypotension, with or without any clinical intervention, to determine if the model predictors were consistent.

## Results

**Fig 1:**
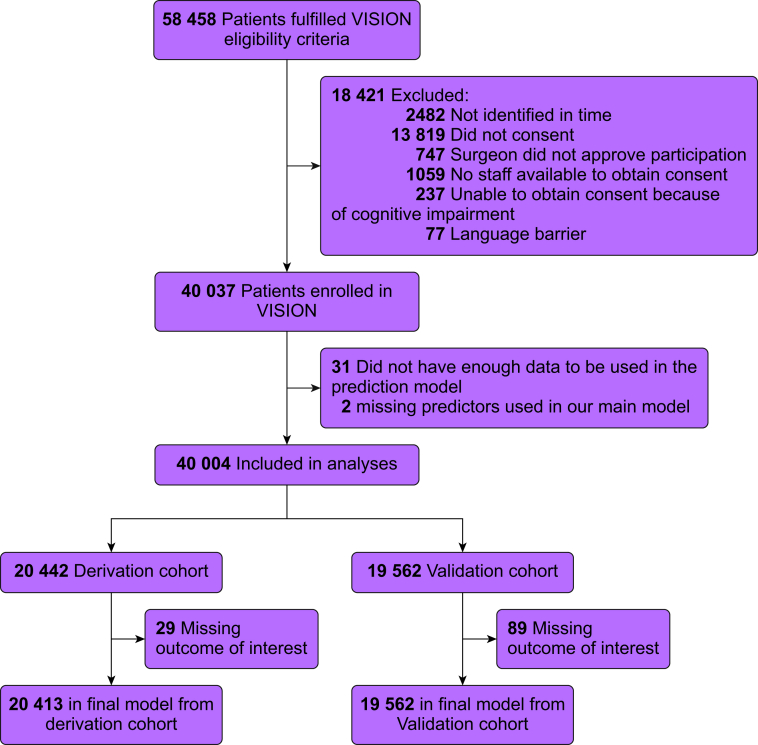
Participant flow.

These analyses included 40 004 patients: 20 442 patients from 14 centres were included in the derivation cohort and 19 562 patients from the other 14 centres were included in the validation cohort (Fig. 1 and Supplementary Tables S1 and S2). Patients had a mean age of 64 yr (Table 1), and half of them were women. The most common comorbidities were hypertension (20 152 patients, 50.5%), active cancer (9832 patients, 24.6%), and diabetes (8332 patients, 20.9%). Most common surgeries were low-risk (14 383 patients, 36.0%), major general surgery (7950, 19.9%), and major orthopaedic surgery (6982 patients, 17.5%). The most common types of anaesthesia were general (29 073 patients, 72.7%) and spinal anaesthesia (9811 patients, 24.5%).

**Table 1:** Cohort characteristics. Clinical characteristics of patients in the derivation cohort *vs* the validation cohort. ADL, activities of daily living; DBP, diastolic blood pressure; EVAR, endovascular aneurysm repair; SBP, systolic blood pressure. ∗*t*-test for heart rate, SBP, and DBP; χ^2^ test for all other variables.

	All patients *N*=40 004	Derivation cohort *N*=20 442	Validation cohort *N*=19 562	*P*-value∗
**Age, mean (range)**	64.0 (44–103)	64.9 (45–102)	63.1 (44–103)	<0.001
**Males, *n* (%)**	20 127 (50.3)	10 113 (49.5)	10 014 (51.2)	0.001
**History of: *n* (%)**				
Smoking	18 643 (46.8) *N*=39 838	9769 (47.8) *N*=20 435	8874 (45.7) *N*=19 403	<0.001
Diabetes	8332 (20.9) *N*=39 905	3939 (19.3) *N*=20 440	4393 (22.6) *N*=19 465	<0.001
Hypertension	20 152 (50.5) *N*=39 917	10 255 (50.2) *N*=20 439	9897 (50.8) *N*=19 478	0.203
Living in a nursing home	574 (1.4) *N*=39 882	342 (1.7)	232 (1.2) *N*=19 440	<0.001
Need assistance with ADL	2130 (5.3) *N*=39 869	896 (4.4) *N*=20 440	1234 (6.4) *N*=19 429	<0.001
History or current atrial fibrillation	1262 (3.2) *N*=39 827	668 (3.3) *N*=20 428	594 (3.1) *N*=19 399	0.236
Congestive heart failure	1424 (3.6) *N*=39 870	702 (3.4) *N*=20 438	722 (3.7) *N*=19 432	0.131
Coronary artery disease	5159 (12.9) *N*=39 876	2560 (12.5) *N*=20 435	2599 (13.4) *N*=19 441	0.012
High-risk coronary artery disease	384 (1.0)	209 (1.0)	175 (0.9)	0.190
Coronary revascularisation over 1 yr	2923 (7.3) *N*=39 869	1486 (7.3) *N*=20 441	1437 (7.4) *N*=19 428	0.627
Coronary revascularisation within 1 yr	564 (1.4) *N*=39 861	310 (1.5) *N*=20 437	254 (1.3) *N*=19 424	0.077
Cardiac arrest	235 (0.6) *N*=39 868	103 (0.5) *N*=20 437	132 (0.7) *N*=19 431	0.022
Aortic stenosis	387 (1.0) *N*=39 861	254 (1.2) *N*=20 433	133 (0.7) *N*=19 428	<0.001
Peripheral vascular disease	3203 (8.0)	1153 (5.6)	2050 (10.5)	<0.001
Cerebral vascular event	2582 (6.5)	1407 (6.9)	1175 (6.0)	<0.001
Deep vein thrombosis/pulmonary embolism	1312 (3.3) *N*=39 865	653 (3.2) *N*=20 439	659 (3.4) *N*=19 426	0.269
Chronic obstructive pulmonary disease	3165 (7.9)	1575 (7.7)	1590 (8.1)	0.117
Obstructive sleep apnoea	1932 (4.9) *N*=39 855	982 (4.8) *N*=20 430	950 (4.9) *N*=19 425	0.697
Active cancer	9832 (24.6)	5287 (25.9)	4545 (23.2) *N*=19 562	<0.001
**Preoperative vital signs, mean (****sd****)**				
Heart rate	77.4 (14.5) *N*=39 716	77.6 (12.6) *N*=20 360	77.2 (14.5) *N*=19 356	0.019
SBP	139.7 (23.3)	141.0 (24.3) *N*=20 399	138.3 (22.2) *N*=19 414	<0.001
DBP	78.6 (13.1)	79.1 (13.5) *N*=20 311	78.1 (12.6) *N*=19 393	<0.001
**Preoperative laboratory tests, mean (****sd****)**				
Estimated glomerular filtration rate	79.6 (24.3) *N*=37 290	78.8 (23.4) *N*=19 022	80.6 (25.1) *N*=18 268	<0.001
Haemoglobin	130.1 (19.3) *N*=38 617	130.5 (19.0) *N*=19 724	129.8 (19.6) *N*=18 893	<0.001
**Surgery, *n* (%)**				
Vascular without EVAR	2352 (5.9)	846 (4.14)	1506 (7.7)	<0.001
EVAR	302 (0.8)	123 (0.6)	179 (0.9)	<0.001
General	7950 (19.9)	4189 (20.5)	3761 (19.2)	0.002
Thoracic	1165 (2.9)	810 (4.0)	355 (1.8)	<0.001
Major urology/gynaecology	4827 (12.1)	2466 (12.1)	2361 (12.1)	0.986
Major orthopaedic	6982 (17.5)	4151 (20.3)	2831 (14.5)	<0.001
Major neurosurgery	2341 (5.9)	1102 (5.4)	1239 (6.3)	<0.001
Low-risk surgeries	15 308 (38.3)	7721 (37.8)	7587 (38.8)	0.037
**Open surgery, *n* (%)**	31 288 (78.3) *N*=39978	15 966 (78.1) *N*=20 439	15 322 (78.4) *N*=19 539	0.464
**Timing of surgery (h), *n* (%)**				<0.001
>72	35 815 (89.5)	17 820 (87.2)	17 995 (92.0)	
24–72	3076 (7.7)	2081 (10.2)	995 (5.1)	
<24	1113 (2.8)	541 (2.7)	572 (2.9)	
**Anaesthesia, *n* (%)**				
General	29 073 (72.7) *N*=39976	14 231 (69.6)	14 842 (76.0) *N*=19 534	<0.001
Spinal	9811 (24.5) *N*=39 971	6053 (29.6)	3758 (19.2) *N*=19 529	<0.001
Epidural	4489 (11.2) *N*=39 968	2213 (10.8) *N*=20 441	2276 (11.7) *N*=19 527	0.009
Nerve block	2761 (6.9) *N*=39 971	982 (4.8)	1779 (9.1) *N*=19 529	<0.001
**Intraoperative hypotension (SBP <90 mm Hg), *n* (%)**	18 487 (47.0) *N*=39 317	8734 (43.6) *N*=20 026	9753 (50.6) *N*=19 291	<0.001
**Postoperative hypotension (SBP <90 mm Hg), *n* (%)**	8733 (21.9) *N*=39 886	4721 (23.1) *N*=20 413	4012 (20.6) *N*=19 473	<0.001

### Incidence of clinically important postoperative hypotension

The incidence of CIPH was 12.4% (4959 patients, 95% CI 12.1–12.8) during the hospitalisation period (Supplementary Table S3). CIPH occurred most frequently on postoperative day 1 (2239 patients, 5.7%, 95% CI 5.5–5.9), in PACU (1632 patients, 4.4%, 95% CI 4.2–4.6), and on the day of surgery after PACU (1379 patients, 3.7%, 95% CI 3.5–3.9).

### Main model

**Fig 2:**
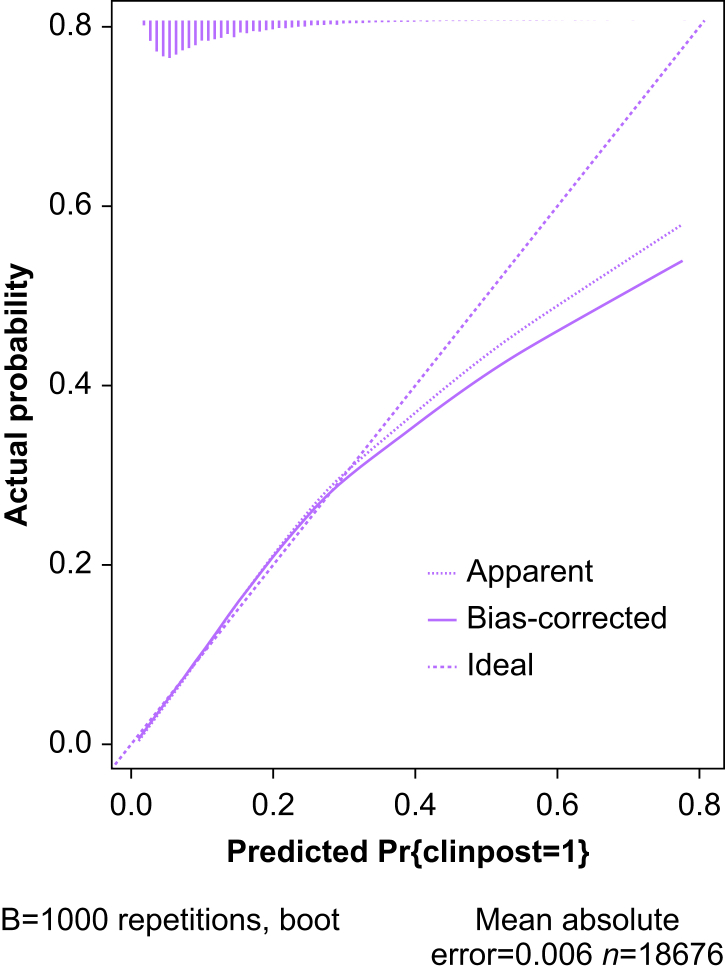
Calibration plot of the main clinical prediction model applied to derivation cohort. Y-axis: observed proportion of patients with clinically important postoperative hypotension. X-axis: predicted risk of clinically important postoperative hypotension. Ideal: line to represent perfect prediction risk equal the observed proportion of patients. Non-parametric: line derived from resampling procedure using bootstrapping technique.

**Fig 3:**
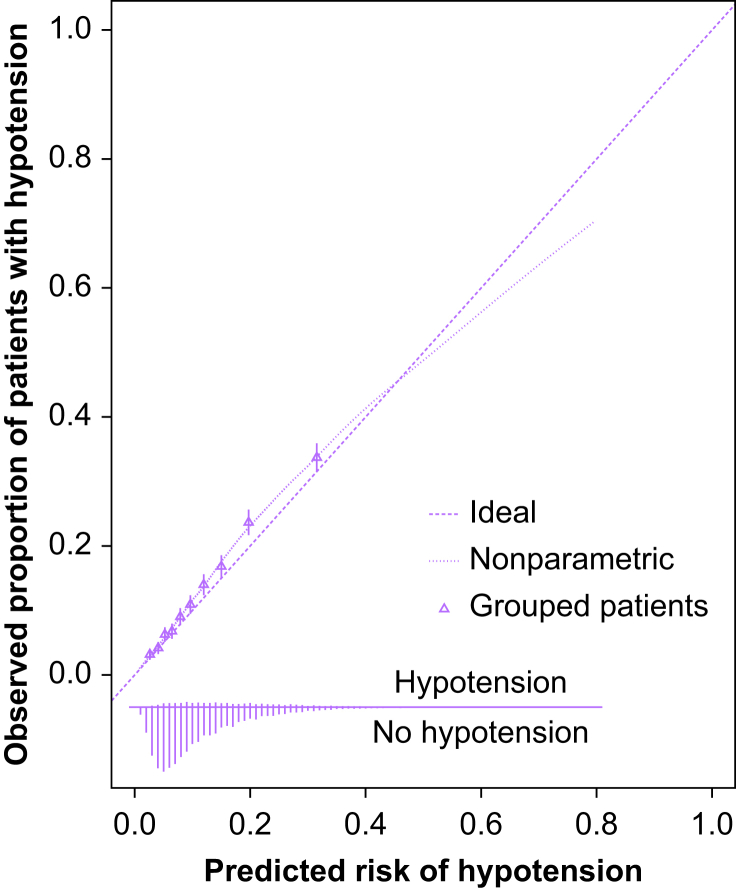
Calibration plot of the main clinical prediction model applied to validation cohort *N*=19 562. Y-axis: observed proportion of patients with clinically important postoperative hypotension. X-axis: predicted risk of clinically important postoperative hypotension. Ideal: line to represent perfect prediction risk equal the observed proportion of patients.

Using a backward elimination procedure, 41 of the 49 candidate predictor variables were retained in the final model. In the derivation cohort, the calibration plot demonstrated that the bias-corrected line closely aligned the ideal line up to 30%, followed by overprediction beyond that value (Fig. 2). In the validation cohort, calibration was maintained with overprediction after 50% (Fig. 3). The model maintained good discrimination (C-statistic in derivation cohort: 0.74, bias-corrected C-statistic in derivation cohort: 0.73, C-statistic in validation cohort: 0.72; Supplementary Table S8). The surgical variables that had the highest risk for postoperative hypotension included specific types of surgeries and open procedures (aOR 2.02; 95% CI 1.73–2.35; Table 2). The surgeries associated with the highest risk of CIPH were pneumonectomy (aOR 5.10; 95% CI 1.92–13.51), complex visceral resection (aOR 4.24; 95% CI 3.42–5.25), and major hip/pelvic surgery (aOR 3.71; 95% CI 3.16–4.36). Significant patient characteristics include history of aortic stenosis (aOR 1.90; 95% CI 1.39–2.58), recent high-risk coronary artery disease (aOR 1.51; 95% CI 1.03–2.19), and history or current atrial fibrillation (aOR 1.42; 95% CI 1.19–1.68). Preoperative SBP <110 mm Hg (aOR 2.47; 95% CI 2.06–2.98), diastolic blood pressure DBP <60 mm Hg (aOR 1.51; 95% CI 1.24–1.83) and preoperative heart rate >100 beats min^−1^ (aOR 1.96; 95% CI 1.55–2.48) were the strongest preoperative hemodynamic predictors of CIPH (Supplementary Table S7).

**Table 2:** Variables included in multivariable logistic regression model for predicting hypotension. Multivariable logistic regression using categorical variables. ADL, activities of daily living; CAD, coronary artery disease; CI, confidence interval; COPD, chronic obstructive pulmonary disease; DBP, diastolic blood pressure; eGFR, estimated glomerular filtration rate; EVAR, endovascular aneurysm repair; OR, odds ratio; SBP, systolic blood pressure; TURP, transurethral resection of the prostate.

Independent variables	Multivariable analysis, *N*=18 676	
	Adjusted OR (95% CI)	*P*-value
**Type of surgery**		
Pneumonectomy	5.10 (1.92–13.51)	0.001
Complex visceral resection	4.24 (3.42–5.25)	<0.001
Major hip/pelvic surgery	3.71 (3.16–4.36)	<0.001
Lobectomy	3.55 (2.60–4.85)	<0.001
Knee arthroplasty	3.32 (2.82–3.89)	<0.001
Radical prostatectomy	3.12 (2.21–4.41)	<0.001
Aorto-iliac reconstructive surgery	3.12 (2.19–4.41)	<0.001
Stomach surgery	2.97 (2.50–3.53)	<0.001
Other thoracic surgery	2.89 (2.17–3.87)	<0.001
Cytoreductive surgery	2.60 (1.75–3.88)	<0.001
Visceral resection	2.43 (1.86–3.16)	<0.001
Radical hysterectomy	2.21 (1.43–3.42)	<0.001
TURP	1.92 (1.34–2.73)	<0.001
Major spine surgery	1.82 (1.36–2.44)	<0.001
Above knee amputation	1.74 (0.89–3.40)	0.106
Head and neck surgery	1.73 (1.18–2.56)	0.005
Craniotomy	1.72 (1.26–2.36)	0.001
Intra-abdominal surgery	1.70 (1.44–2.01)	<0.001
Internal fixation of femur	1.51 (1.08–2.12)	0.015
Hysterectomy	1.50 (1.16–1.94)	0.002
EVAR	1.42 (0.70–2.86)	0.330
Peripheral vascular reconstruction	1.42 (0.99–2.07)	0.057
Thoracic aorta reconstructive surgery	1.27 (0.54–2.98)	0.579
Cerebrovascular surgery	1.18 (0.71–1.94)	0.527
Lower leg amputation	0.99 (0.44–2.22)	0.984
Open *vs* endoscopic surgery	2.02 (1.73–2.35)	<0.001
**Timing of surgery (h)**		
>72	Reference	
24–72	0.69 (0.58–0.83)	<0.001
<24	1.27 (0.97–1.68)	0.082
**Patient characteristics**		
Age (yr)		
45–49	Reference	
50–64	1.22 (1.00–1.48)	0.050
65–79	1.36 (1.10–1.68)	0.004
≥80	1.35 (1.05–1.72)	0.017
Male sex	0.76 (0.69–0.85)	<0.001
History of smoking	1.27 (1.15–1.40)	<0.001
Need assistance with ADL	1.37 (1.13–1.67)	0.002
History or current atrial fibrillation	1.42 (1.19–1.68)	<0.001
**Coronary artery disease**		
No CAD	Reference	
History of CAD	1.25 (1.08–1.43)	0.002
Recent high-risk CAD	1.51 (1.03–2.19)	0.031
**History of aortic stenosis**	1.88 (1.38–2.56)	<0.001
**History of COPD**	1.39 (1.19–1.62)	<0.001
**History of diabetes mellitus**	0.86 (0.76–0.97)	0.016
**Preoperative haemodynamics**		
Preoperative SBP (mm Hg)		
<110	2.47 (2.06–2.98)	<0.001
110–139	1.49 (1.33–1.67)	<0.001
140–174	Reference	
≥175	0.78 (0.64–0.94)	0.009
Preoperative DBP (mm Hg)		
<60	1.51 (1.24–1.83)	<0.001
60–79	1.31 (1.17–1.46)	<0.001
80–99	Reference	
≥100	0.90 (0.72–1.13)	0.389
Preoperative heart rate (beats min^−1^)		
<60	Reference	
60–79	1.19 (0.99–1.43)	0.063
80–99	1.33 (1.10–1.61)	0.004
≥100	1.96 (1.55–2.48)	<0.001
**Preoperative laboratory values**		
Haemoglobin (g L^−1^)		
>150	Reference	
130–150	1.13 (0.97–1.32)	0.115
100–129	1.27 (1.08–1.49)	0.003
<100	1.21 (0.95–1.51)	0.120
eGFR (ml min^−1^ 1.73 m^−2^)		
>100	Reference	
80–99	1.03 (0.88–1.21)	0.684
45–79	1.14 (0.96–1.34)	0.127
Dialysis–44	1.28 (1.03–1.58)	0.023

### Simplified prediction model

The variables in the simplified model were high-risk surgery (a score of 5), open surgery (a score of 4), SBP <130 mm Hg (a score of 3), and heart rate >100 beats min^−1^ (a score of 2) (Table 3 and Supplementary Tables S5 and S6). This simplified model had fair discrimination (C-statistic in derivation cohort: 0.68, bias-corrected C-statistic: 0.68, C-statistic in validation cohort: 0.68) (Supplementary Table S8) and calibration was maintained throughout all probabilities in the derivation cohort (Supplementary Fig. S1). In the validation cohort, calibration was also maintained up to 40% (Supplementary Fig. S2).

**Table 3:** Simplified model with risk score. ∗High-risk surgery include: major hip/pelvic surgery (hemi or total hip arthroplasty, internal fixation of hip, pelvic arthroplasty), knee arthroplasty, complex visceral resection (surgery involving the liver, oesophagus, pancreas, or multiple organs), stomach surgery, lobectomy, other thoracic surgeries (wedge resection of lung, resection of mediastinal tumour, major chest wall resection), visceral resection (nephrectomy, ureterectomy, bladder resection, retroperitoneal tumour resection, exenteration), radical prostatectomy, aorto-iliac reconstruction.

Variable	Categories	Odds ratio	Points
High-risk surgery∗	Yes	2.88	5
	No	1.00	0
Open surgery	Yes	2.18	4
	No	1.00	0
Preoperative systolic blood pressure <130 mm Hg	Yes	1.85	3
	No	1.00	0
Preoperative heart rate >100 beats min^−1^	Yes	1.56	2
	No	1.00	0

### Sensitivity analyses

A model including the preoperative variables and intraoperative variables was derived. The intraoperative variables included intraoperative hypotension (SBP <90 mm Hg), intraoperative tachycardia (heart rate >100 beats min^−1^), intraoperative bradycardia (heart rate <55 beats min^−1^), and surgical time. This model maintained calibration up to 30% with overprediction beyond this value (Supplementary Figs S3 and S4). In the validation cohort, similar miscalibration occurred beyond 30% with an overprediction of risk. This model's ability to discriminate was slightly better than the main model using only preoperative variables (C-statistics in derivation cohort: 0.75, C-statistics bias-corrected: 0.75, C-statistics in validation cohort 0.73; Supplementary Table S8).

A secondary model was created using the preoperative variables in the main model and the two most common antihypertensive agents: angiotensin-converting enzyme inhibitors/angiotensin receptor blockers and beta-blockers. This model did not improve the calibration (Supplementary Figs S5 and S6) or discrimination (C-statistics in derivation cohort: 0.74, C-statistics bias-corrected: 0.73, C-statistics in validation cohort: 0.73; Supplementary Table S8). Overprediction occurred above 30% in the derivation cohort and above 45% in the validation cohort. Similar results were found with the calibration of the main model on the imputed data (Supplementary Figs S7 and S8), and discrimination (C-statistics of derivation cohort: 0.74, C-statistics bias-corrected: 0.73, C-statistics of validation cohort: 0.73; Supplementary Table S8). Overprediction occurred above 30% in the derivation cohort and above 50% in the validation cohort. A multivariable logistic regression model with the outcome of postoperative hypotension with or without intervention demonstrated that almost all predictors remained statistically significant except three predictors: age, preoperative heart rate, and preoperative estimated glomerular filtration rate.

## Discussion

### Principal findings

This study demonstrated that CIPH can be predicted using preoperative patient characteristics and surgical information with moderate discrimination and acceptable calibration. However, overprediction occurred around 30%. This model contains 41 variables, with 25 related to surgery type. These variables represent 15 information items that are all readily available in the routine care of a surgical patient. This reflects the complexity and variability of preoperative factors contributing to CIPH. Although the model's performance is modest, its strength lies in identifying key predictors of CIPH and providing a basis for future refinement and validation efforts.

We also developed a simplified model that was created using the following four variables: high-risk surgery, open surgery, preoperative SBP <130 mm Hg, and preoperative heart rate >100 beats min^−1^. The simplified model offers a rapid bedside assessment. Although this model is useful for broad risk stratification, while its C-statistics are lower than the primary model, it still provides clinically relevant discrimination, making it a useful tool as an initial screening in a resource-limited or time-limited environment. It is important to recognise that the full model remains the preferred approach as it enables a more refined risk prediction and individualised clinical decision-making.

### Strengths and weaknesses

Our study has several strengths. First, this is the largest observational study to date to assess CIPH in noncardiac surgery patients. Second, the inclusion of surgical patients who underwent noncardiac surgery from 28 centres and 14 countries worldwide maximised the external validity of our findings. Third, the quality of the data collection and statistical methods were rigorous, and the latter were prespecified. Fourth, there was minimal missing data with near-complete 30-day follow-up.

One important limitation of our analysis is that the detection of CIPH was based on routine practice. On the surgical ward, vital signs are typically recorded at intervals of 4–8 h.^15^ Previous studies suggest that hypotensive episodes may have been missed because of this routine monitoring schedule.^16^^,^^17^ In contrast, monitoring in the ICU or PACU is conducted more frequently. The possibility exists that the increased frequency of monitoring in the ICU led to a higher detection rate of CIPH, introducing a potential detection bias. A future prospective study with continuous vital sign monitoring is needed to externally validate this data.

Another important limitation includes the fact that the last VISION patient was observed in 2014; changes in clinical practice, patient characteristics, and surgical techniques may have occurred over the past decade. Significant advances in surgical technique, perioperative protocols, patient management strategies (e.g. adoption of minimally invasive surgery and enhanced recovery after surgery protocol), and refined approaches to vasopressor and fluid management have become standard practice.^18^ Nonetheless, the two variables in the simplified model, high-risk surgeries and open surgeries, remain relevant today. A recent study analysing a cohort of 23 000 colorectal patients reported that 81% of these procedures were completed using an open approach.^19^ Additionally, the high-risk surgeries listed in our simplified model continue to be prevalent. For instance, total hip and knee arthroplasties, both classified as high-risk surgeries, are projected to increase significantly by 139–176% in the upcoming years.^20^ While some changes could impact the direct applicability of our model, the fundamental risk factors for CIPH remain relevant, and our findings provide a foundation for future external validation using contemporary data.

### Comparison with other studies

A previous study that examined risk factors for postoperative hypotension found similar results. In an observational cohort study of 1588 patients who underwent total hip or knee arthroplasty, female sex was associated with a significantly increased risk of postoperative hypotension. That study also identified a history of stroke, and the use of intraoperative dexmedetomidine as risk factors.^21^ Other previous clinical prediction models examined intraoperative hypotension. In a prospective cohort study of 193 patients who had noncardiac surgery, preoperative hypotension, advanced age, and major surgery were risk factors for intraoperative hypotension.^22^ In a large retrospective cohort study of 58 458 patients undergoing noncardiac surgery, the investigators examined the risk factors for intraoperative cardiovascular events. This study identified that the ASA physical status classification score and revised cardiac risk index (RCRI) were predictive for intraoperative cardiovascular events.^23^

### Explanation and implications

Although there are numerous definitions of perioperative hypotension in the literature,^4^ we selected CIPH as the outcome of interest because it reflects real-world clinical decision-making and the need for intervention. Most clinicians would intervene for a surgical patient with an SBP <90 mm Hg regardless of the patient's underlying characteristics, except in specific cases (e.g. underlying cirrhosis, or heart failure with reduced ejection fraction).^24^^,^^25^ We acknowledge that our outcome definition may introduce selection bias, as treatment decisions could influence the observed incidence of CIPH. However, our sensitivity analysis demonstrated that nearly all the predictors remained consistent for the outcome of postoperative hypotension regardless of whether an intervention was performed. This suggests that clinicians generally adhere to similar management practices, and the predictive value of our model is unlikely to be significantly distorted by treatment-related selection bias.

The primary objective of our model is not to replace other established tools, such as American College of Surgeon's National Surgical Quality Improvement Program (ACS NSQIP) surgical risk calculator, which estimates complications or death after surgery.^26^ Our model provides a complementary predictive capability focusing on postoperative haemodynamics. CIPH is an early postoperative event that can lead to prolonged recovery, increased complications, and higher resource utilisation. Although our model predicts an intermediate variable rather than a more distal clinical outcome, CIPH represents a modifiable and immediate risk factor that directly influences postoperative outcomes. Predicting CIPH in the preoperative setting offers distinct advantages by enabling targeted postoperative interventions, such as anticipating fluid requirements, individualised vasopressor support, or closer postoperative monitoring.

The current practice to determine the disposition of high-risk patients to the ICU is mainly based on high-risk surgery and intraoperative factors (e.g. vasopressor use, blood loss).^27^ For the highest-risk patients, clinicians can adjust monitoring frequency, anticipate fluid and vasopressor administration, and determine appropriate patient disposition, such as ICU, prolonged stays in the PACU, and utilisation of remote automated monitoring technology.^28^ By identifying the patients at higher risk of CIPH, healthcare systems can ensure targeted resource allocation by increasing monitoring or optimising staffing in high-acuity settings. The proactive approach assists with timely intervention, potentially averting serious complications, and ensures optimal resource utilisation. However, external validation must confirm its generalisability and ensure reliable performance in different clinical settings. This study provides a robust foundation for future external validation efforts, as the well-defined predictors identified here could streamline subsequent validation studies and reduce the required sample size.

## Conclusions

In summary, internal–external validation of this large international, prospective cohort study demonstrated that CIPH, defined as SBP <90 mm Hg that required a clinical intervention, can be predicted before surgery using our primary model and a simple four-element model. This model can help clinicians determine patient disposition and monitoring strategies. However, a prospective study with a continuous blood pressure monitor is needed to externally validate this prediction model.

## Authors’ contributions

Study concept/design: all authors

Data acquisition/analysis: all authors

Data interpretation: SSY, PSR, DHA, PJD

Article drafting: SSY, PJD

Revising the draft critically for important intellectual content: all authors

Final approval of the version to be published: all authors

Accountable for all aspects of the work: all authors

## Funding

VISION. Canada: 10.13039/501100000024Canadian Institutes of Health Research; Heart and Stroke Foundation of Ontario; Academic Health Science Centres Alternative Funding Plan Innovation Fund Ontario; 10.13039/100030936Population Health Research Institute; CLARITY Research Group; McMaster University Department of Surgery Surgical Associates; Hamilton Health Science New Investigator Fund; 10.13039/100008360Hamilton Health Sciences; Ontario Ministry of Resource and Innovation; Stryker Canada; 10.13039/100009776McMaster University, Department of Anesthesiology; St Joseph’s Healthcare, Department of Medicine; Father Sean O’Sullivan Research Centre; McMaster University Department of Medicine; Roche-Diagnostics Global Office; Hamilton Health Sciences Summer Studentships; McMaster University Department of Clinical Epidemiology and Biostatistics; 10.13039/100009776McMaster University, Division of Cardiology; Canadian Network and Centre for Trials Internationally; Winnipeg Health Sciences Foundation; University of Manitoba Department of Surgery; Diagnostic Services of Manitoba Research; 10.13039/100008795Manitoba Medical Services Foundation; 10.13039/100008793Manitoba Health Research Council; University of Manitoba Faculty of Dentistry Operational Fund; University of Manitoba Department of Anesthesia; University Medical Group, 10.13039/100011933Department of Surgery, University of Manitoba, Start-up Fund. Australia: National Health and Medical Research Council Program. Brazil: Projeto Hospitais de Excelência a Serviço do SUS (PROADI-SUS), Brazilian Ministry of Health, Hcor (Cardiac Hospital Sao Paulo); 10.13039/501100003593National Council for Scientific and Technological Development, Brazilian Ministry of Science and Technology. China: Public Policy Research Fund (CUHK-4002-PPR-3), Research Grant Council, Hong Kong SAR; General Research Fund (461412), Research Grant Council, Hong Kong SAR; 10.13039/501100001136Australian and New Zealand College of Anaesthetists (13/008). Colombia: School of Nursing, Universidad Industrial de Santander; Grupo de Cardiología Preventiva, Universidad Autónoma de Bucaramanga; 10.13039/501100008727Fundación Cardioinfantil - Instituto de Cardiología; Alianza Diagnóstica SA. France: 10.13039/501100005737Université Pierre et Marie Curie, Département d’anesthésie Réanimation, Pitié-Salpêtrière, Assistance Publique–Hôpitaux de Paris. India: St John’s Medical College and Research Institute; Division of Clinical Research and Training. Malaysia: 10.13039/501100004386University of Malaya (grant RG302-14AFR); University of Malaya, Penyelidikan Jangka Pendek. Poland: Polish Ministry of Science and Higher Education (NN402083939). South Africa: 10.13039/501100004695University of KwaZulu-Natal. Spain: Instituto de Salud Carlos III; Fundació La Marató de TV3. USA: 10.13039/100000968American Heart Association; Covidien. UK: 10.13039/501100000272National Institute for Health Research.

## Declarations of interest

Roche-Diagnostics provided Troponin T assays and some financial support for the study. PJD reports grants from Roche-Diagnostics and Abbott-Diagnostics during the conduct of the study, and grants from Octapharma, Philips Healthcare, Stryker, Covidien, and Boehringer Ingelheim outside the submitted work. The remaining authors note no relationships or activities that could appear to have influenced the submitted work.
