# Supplementary material for: Derivation and internal–external validation of clinical prediction model for postoperative clinically important hypotension in patients undergoing noncardiac surgery: an international prospective cohort study
Source: BJA Open. 2025 May 22;14:100410. doi: 10.1016/j.bjao.2025.100410 (PMC12150106; doi:10.1016/j.bjao.2025.100410)
Supplement: Multimedia component 1 [file mmc1.docx]

Derivation and internal-external validation of clinical prediction model for postoperative clinically important hypotension in patients undergoing noncardiac surgery: an international prospective cohort study

**Supplemental Digital Content**

**Table of Contents**

[Supplementary Table S1: Recruitment by country and centre 2](#_Toc197429494)

[Supplementary Table S2: Centers in derivation or validation cohort 4](#_Toc197429495)

[Supplementary Table S3: Timing of postoperative hypotension 5](#_Toc197429496)

[Supplementary Table S4: Variance inflation factor of predictors included in main model 6](#_Toc197429497)

[Supplementary Table S5: Wald test of predictors in the final model 7](#_Toc197429498)

[Supplementary Table S6: Simplified score and predicted probability of postoperative CIH 8](#_Toc197429499)

[Supplementary Table S7: Missing data in the derivation cohort 9](#_Toc197429500)

[Supplementary Table S8: C-statistics of various models 10](#_Toc197429501)

[Supplementary Figure S1: Calibration plot of simplified model in derivation cohort 11](#_Toc197429502)

[Supplementary Figure S2: Calibration plot of simplified model in validation cohort 12](#_Toc197429503)

[Supplementary Figure S3: Calibration plot of model including intraoperative variables in derivation cohort 13](#_Toc197429504)

[Supplementary Figure S4: Calibration plot of model including intraoperative variables in validation cohort 14](#_Toc197429505)

[Supplementary Figure S5: Calibration plot of model including antihypertensive medications in derivation cohort 15](#_Toc197429506)

[Supplementary Figure S6: Calibration plot of model including antihypertensive medications in validation cohort 16](#_Toc197429507)

[Supplementary Figure S7: Calibration plot of model using imputation in derivation cohort 17](#_Toc197429508)

[Supplementary Figure S8: Calibration plot of model using imputation in validation cohort 18](#_Toc197429509)

[Supplementary variable definitions 19](#_Toc197429510)

[Surgical Variables 19](#_Toc197429511)

[Patient characteristics 19](#_Toc197429512)

# Supplementary Table S1: Recruitment by country and centre

| Continent, country, city, centre | Participants  (n=40,004) |
| --- | --- |
| North America  Canada  *Hamilton*  Juravinski Hospital and Cancer Centre  Saint Joseph’s Healthcare  Hamilton General Hospital  McMaster University Medical Centre  *Winnipeg*  Health Sciences Centre Winnipeg  *Edmonton*  Walter C. MacKenzie Health Sciences Centre  *London*  Victoria Hospital  United States  *Cleveland*  Cleveland Clinic  *St. Louis*  Washington University School of Medicine | (11,693)  3884  1003  751  642  1697  1580  747  1248  141 |
| Asia  China  *Hong Kong*  Prince of Wales Hospital  India  *Bangalore*  St. John’s Medical College Hospital  *Ludhiana*  Christian Medical College  Malaysia  *Kuala Lumpur*  University Malaya Medical Centre | (10,005)  4413  1996  1549  2047 |
| Europe  United Kingdom  *London*  Barts And The London  University College Hospital  *Leeds*  Leeds Teaching Hospitals  *Liverpool*  Royal Liverpool University Hospital  Spain  *Barcelona*  Hospital de Sant Pau  *Madrid*  Hospital Gregorio Maranon  Poland  *Krakow*  Jagiellonian University Medical College  France  *Paris*  Pitie-Salpetriere Hospital | (9671)  2007  880  733  722  1985  1764  982  598 |
| South America  Brazil  *São Paulo*  Hospital do Coracao  *Porto Alegre*  Hospital de Clinicas de Porto Alegre  Colombia  *Bucaramanga*  Hospital Universitario de Santander  *Bogota*  Foundation CardioInfanil  Peru  *Lima*  Hospital Nacional Cayetano Heredia | (6063)  1503  1001  1392  628  1539 |
| Africa  South Africa  *Durban*  Inkosi Albert Luthuli Hospital | (1489)  1489 |
| Australia  Australia  *Sydney*  Westmead Hospital | (1083)  1083 |

# Supplementary Table S2: Centers in derivation or validation cohort

| Derivation cohort centers (n) | Validation cohort centers (n) |
| --- | --- |
| Winnipeg, Canada (1697) | Cleveland, OH, USA (1248) |
| Paris, France (598) | Krakow, Poland (982) |
| Bogota, Colombia (628) | Porto Alegre, Brazil (1001) |
| Hamilton General Hospital, Canada (751) | Liverpool, UK (722) |
| Henderson/Juravinski Hospital, Canada (3884) | UCH London, UK (880) |
| Victoria Hospital, Canada(747) | Saint Joseph’s Healthcare Hamilton, Canada (1003) |
| McMaster University Medical Centre, Canada (642) | Edmonton, Canada (1580) |
| India Christian, Canada (1549) | St-John, India (1996) |
| Bucaramanga, Colombia (1392) | Lima, Peru (1539) |
| Sao Paulo, Brazil (1503) | Inkosi Albert Luthuli Hospital, South Africa (1489) |
| Madrid, Spain (1764) | Barcelona, Spain (1985) |
| Hong Kong, China (4413) | Kuala Lumpur, Malaysia (2047) |
| Leeds, UK (733) | London, UK (2007) |
| St. Louis, Missouri, USA (141) | Westmead Hospital, Sydney, Australia (1083) |

# Supplementary Table S3: Timing of postoperative hypotension

| Timing | Patients with clinically important hypotension, No. (%) | 95% CI^*^ |
| --- | --- | --- |
| In PACU | 1632 (4.4)  N=36967 | 4.2-4.6 |
| Day of surgery post-PACU | 1379 (3.7)  N=37178 | 3.5-3.9 |
| Day 1 after surgery | 2239 (5.7)  N=39380 | 5.5-5.9 |
| Day 2 after surgery | 841 (2.5)  N=34156 | 2.3-2.6 |
| Day 3 after surgery | 383 (1.3)  N=30148 | 1.1-1.4 |
| Later than day 3 after surgery | 668 (2.5)  N=26863 | 2.3-2.7 |
| From PACU until discharged | 4959 (12.4)  N=39886 | 12.1-12.8 |

Results expressed as n (%) unless otherwise stated. CI: confidence interval; PACU: post-anesthetic care unit.

^*^95% confidence interval calculated using normal approximation

# Supplementary Table S4: Variance inflation factor of predictors included in main model

| Variable | VIF |
| --- | --- |
| EVAR | 1.03 |
| Thoracic aorta reconstructive surgery | 1.01 |
| Aorto-iliac reconstructive surgery | 1.05 |
| Peripheral vascular reconstruction | 1.07 |
| Cerebrovascular surgery | 1.05 |
| Complex visceral resection | 1.04 |
| Stomach surgery | 1.09 |
| Intra-abdominal surgery | 1.14 |
| Head and neck surgery | 1.03 |
| Pneumonectomy | 1.00 |
| Lobectomy | 1.05 |
| Other thoracic surgery | 1.05 |
| Visceral resection | 1.03 |
| Cytoreductive surgery | 1.03 |
| Hysterectomy | 1.11 |
| Radical hysterectomy | 1.03 |
| Radical prostatectomy | 1.05 |
| TURP | 1.17 |
| Major hip/pelvic surgery | 1.24 |
| Internal fixation of femur | 1.14 |
| Knee arthroplasty | 1.26 |
| Above knee amputation | 1.03 |
| Lower leg amputation | 1.03 |
| Craniotomy | 1.07 |
| Major spine surgery | 1.07 |
| Open surgery | 1.28 |
| Timing of surgery  24-72 hours  < 24 hours | 1.14  1.03 |
| Age | 1.63 |
| Male sex | 1.30 |
| History of smoking | 1.18 |
| Need assistance with ADL | 1.08 |
| History or current atrial fibrillation | 1.08 |
| History of COPD | 1.09 |
| History of diabetes mellitus | 1.07 |
| Coronary Artery disease  History of CAD  Recent high-risk CAD | 1.12  1.03 |
| History of aortic stenosis | 1.03 |
| Preoperative systolic blood pressure | 1.72 |
| Preoperative diastolic blood pressure | 1.70 |
| Preoperative HR | 1.13 |
| Preoperative eGFR | 1.35 |
| Preoperative hemoglobin | 1.24 |
| Mean VIF | **1.14** |

ADL: activities of daily living; CAD: coronary artery disease; COPD: chronic obstructive pulmonary disease; eGFR: estimated glomerular filtration rate; EVAR: endovascular aneurysm repair; HR: heart rate; TURP: transurethral resection of the prostate; VIF: variance inflation factor

# Supplementary Table S5: Wald test of predictors in the final model

| Variable | χ^2^ | df | p-value | Included in the simplified model |
| --- | --- | --- | --- | --- |
| Major hip/pelvic surgery | 259.38 | 1 | <0.001 | Yes |
| Knee arthroplasty | 215.95 | 1 | <0.001 | Yes |
| Complex visceral resection | 173.90 | 1 | <0.001 | Yes |
| Stomach surgery | 147.93 | 1 | <0.001 | Yes |
| Preoperative systolic blood pressure | 117.08 | 2 | <0.001 | Yes |
| Open surgery | 78.68 | 1 | <0.001 | Yes |
| Lobectomy | 60.53 | 1 | <0.001 | Yes |
| Other thoracic surgery | 53.53 | 1 | <0.001 | Yes |
| Visceral resection | 45.62 | 1 | <0.001 | Yes |
| Radical prostatectomy | 41.80 | 1 | <0.001 | Yes |
| Aorto-iliac reconstruction | 41.00 | 1 | <0.001 | Yes |
| Preoperative heart rate | 41.30 | 2 | <0.001 | Yes |
| Intra-abdominal surgery | 38.02 | 1 | <0.001 | No |
| Male sex | 26.97 | 1 | <0.001 | No |
| Preoperative diastolic blood pressure | 24.21 | 2 | <0.001 | No |

df: degrees of freedom

# Supplementary Table S6: Simplified score and predicted probability of postoperative CIH

| Score | Probability (%) |
| --- | --- |
| 0 | 3.4 |
| 2 | 5.0 |
| 3 | 6.0 |
| 4 | 7.3 |
| 5 | 8.7 |
| 6 | 10.5 |
| 7 | 12.5 |
| 8 | 14.8 |
| 9 | 17.5 |
| 10 | 20.6 |
| 11 | 24.1 |
| 12 | 27.9 |
| 13 | 32.1 |
| 14 | 36.6 |

# Supplementary Table S7: Missing data in the derivation cohort

| Variable | No. of patients with missing data |
| --- | --- |
| Thoracic aortic reconstruction | 3 |
| Open surgery | 2 |
| History of smoking | 7 |
| Patient need assistance with ADL | 2 |
| History of aortic stenosis | 9 |
| History of coronary artery disease | 7 |
| History of hypertension | 3 |
| History of diabetes mellitus | 2 |
| Preoperative SBP | 32 |
| Preoperative DBP | 120 |
| Preoperative HR | 70 |
| Preoperative eGFR | 1418 |
| Preoperative hemoglobin | 716 |

ADL: activities of daily living; DBP: diastolic blood pressure; eGFR: estimated glomerular filtration rate; HR: heart rate; SBP: systolic blood pressure

# Supplementary Table S8: C-statistics of various models

| Prediction model | Development cohort | Development cohort (optimism corrected) | Validation cohort |
| --- | --- | --- | --- |
| Main model | 0.73 | 0.73 | 0.72 |
| Simplified model | 0.68 | 0.68 | 0.68 |
| Model including intraoperative variables | 0.75 | 0.75 | 0.73 |
| Model including antihypertensive drugs | 0.74 | 0.73 | 0.73 |
| Model using imputation | 0.74 | 0.73 | 0.73 |

# Supplementary Figure S1: Calibration plot of simplified model in derivation cohort


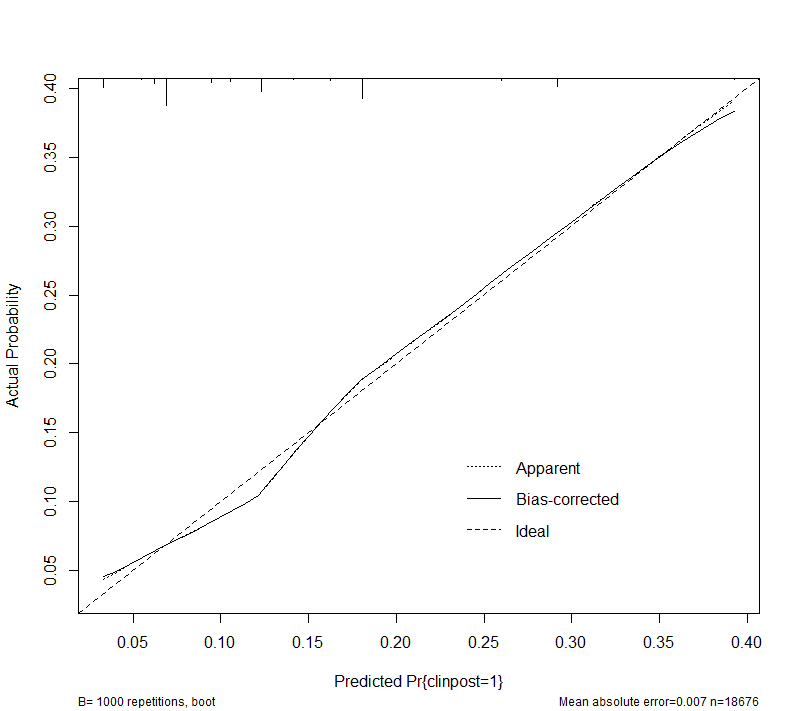


Calibration plot of the simplified clinical prediction model applied to derivation cohort. Y-axis: observed proportion of patients with clinically important postoperative hypotension. X-axis: predicted risk of clinically important postoperative hypotension. Ideal: line to represent perfect prediction risk equal the observed proportion of patients. Nonparametric: line derived from resampling procedure using bootstrapping technique.

# Supplementary Figure S2: Calibration plot of simplified model in validation cohort


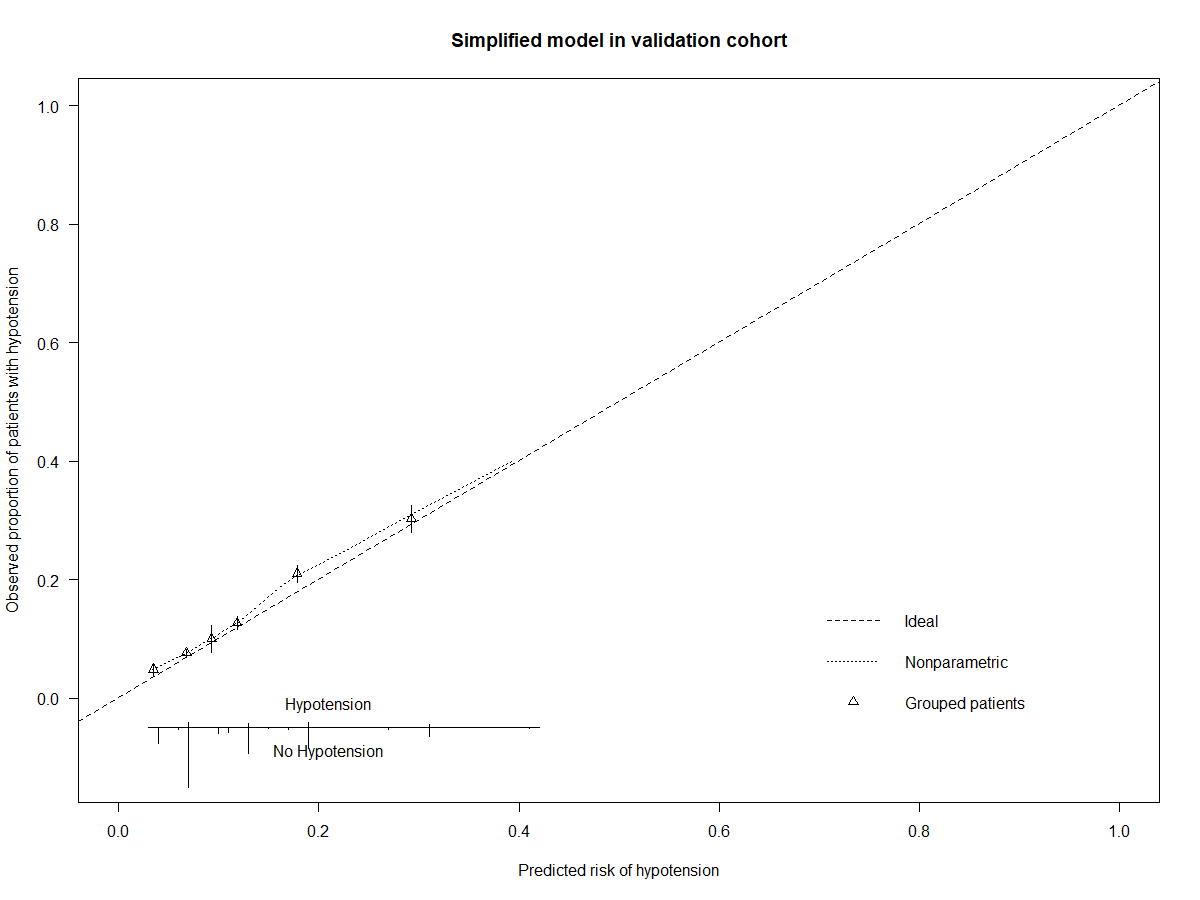


Calibration plot of the simplified clinical prediction model applied to validation cohort. Y-axis: observed proportion of patients with clinically important postoperative hypotension. X-axis: predicted risk of clinically important postoperative hypotension. Ideal: line to represent perfect prediction risk equal the observed proportion of patients.

# Supplementary Figure S3: Calibration plot of model including intraoperative variables in derivation cohort


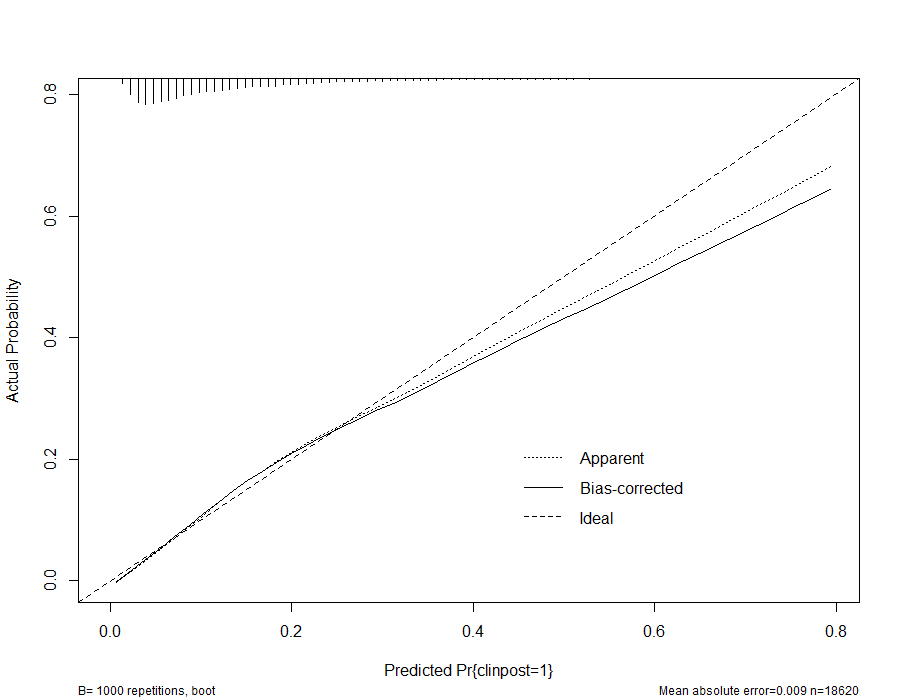


Calibration plot of the clinical prediction model including intraoperative variables applied to derivation cohort. Y-axis: observed proportion of patients with clinically important postoperative hypotension. X-axis: predicted risk of clinically important postoperative hypotension. Ideal: line to represent perfect prediction risk equal the observed proportion of patients. Nonparametric: line derived from resampling procedure using bootstrapping technique.

# Supplementary Figure S4: Calibration plot of model including intraoperative variables in validation cohort


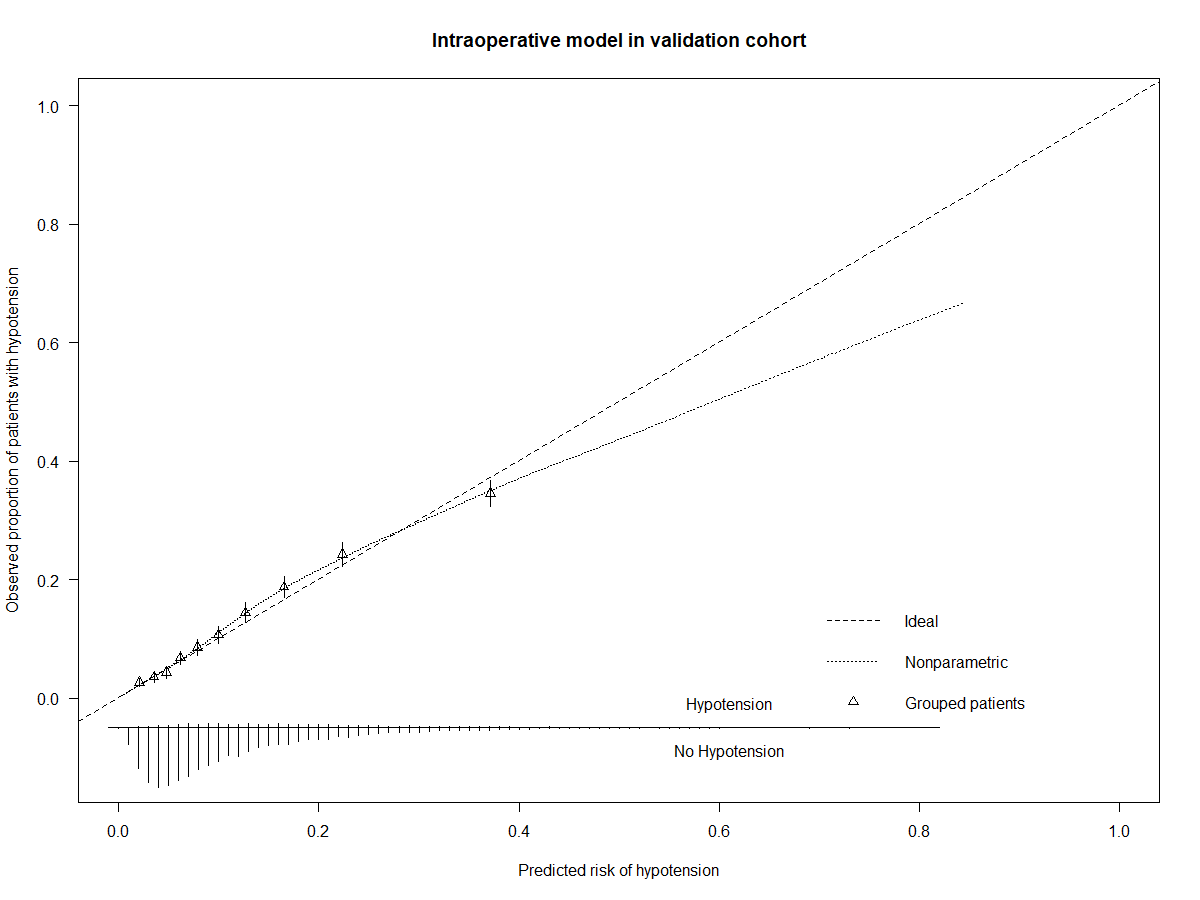


Calibration plot of the clinical prediction model including intraoperative variables applied to validation cohort. Y-axis: observed proportion of patients with clinically important postoperative hypotension. X-axis: predicted risk of clinically important postoperative hypotension. Ideal: line to represent perfect prediction risk equal the observed proportion of patients.

# Supplementary Figure S5: Calibration plot of model including antihypertensive medications in derivation cohort


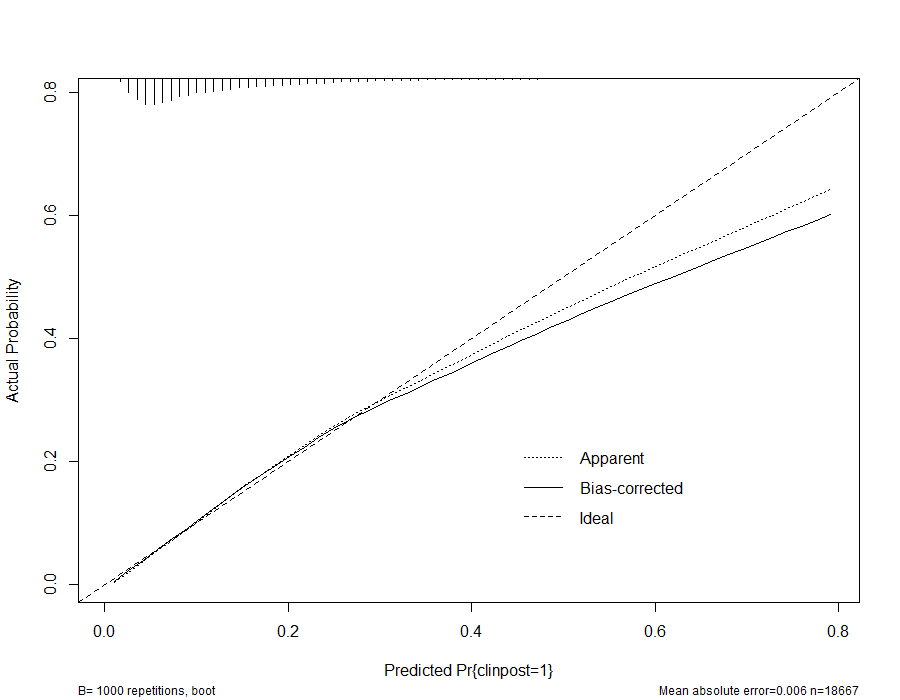


Calibration plot of the clinical prediction model including antihypertensive medication variables applied to derivation cohort. Y-axis: observed proportion of patients with clinically important postoperative hypotension. X-axis: predicted risk of clinically important postoperative hypotension. Ideal: line to represent perfect prediction risk equal the observed proportion of patients. Nonparametric: line derived from resampling procedure using bootstrapping technique.

# Supplementary Figure S6: Calibration plot of model including antihypertensive medications in validation cohort


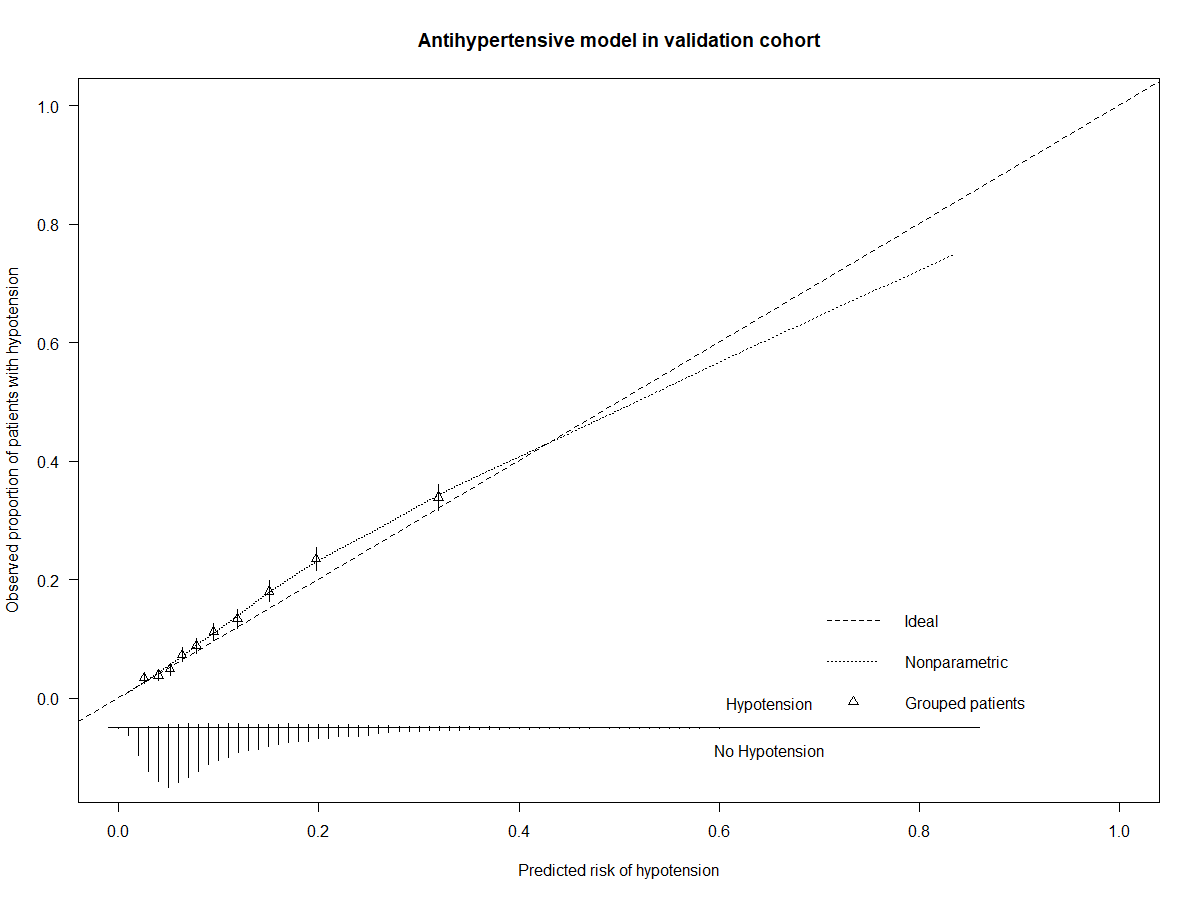


Calibration plot of the clinical prediction model including antihypertensive medication variables applied to validation cohort. Y-axis: observed proportion of patients with clinically important postoperative hypotension. X-axis: predicted risk of clinically important postoperative hypotension. Ideal: line to represent perfect prediction risk equal the observed proportion of patients.

# Supplementary Figure S7: Calibration plot of model using imputation in derivation cohort


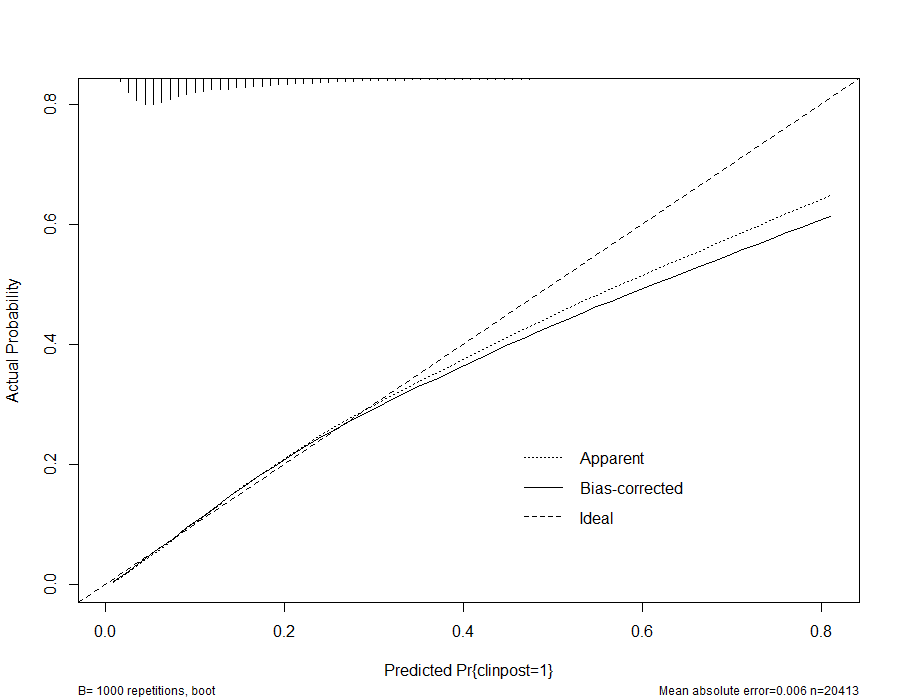


Calibration plot of the clinical prediction model using simple stochastic imputation applied to derivation cohort. Y-axis: observed proportion of patients with clinically important postoperative hypotension. X-axis: predicted risk of clinically important postoperative hypotension. Ideal: line to represent perfect prediction risk equal the observed proportion of patients. Nonparametric: line derived from resampling procedure using bootstrapping technique.

# Supplementary Figure S8: Calibration plot of model using imputation in validation cohort


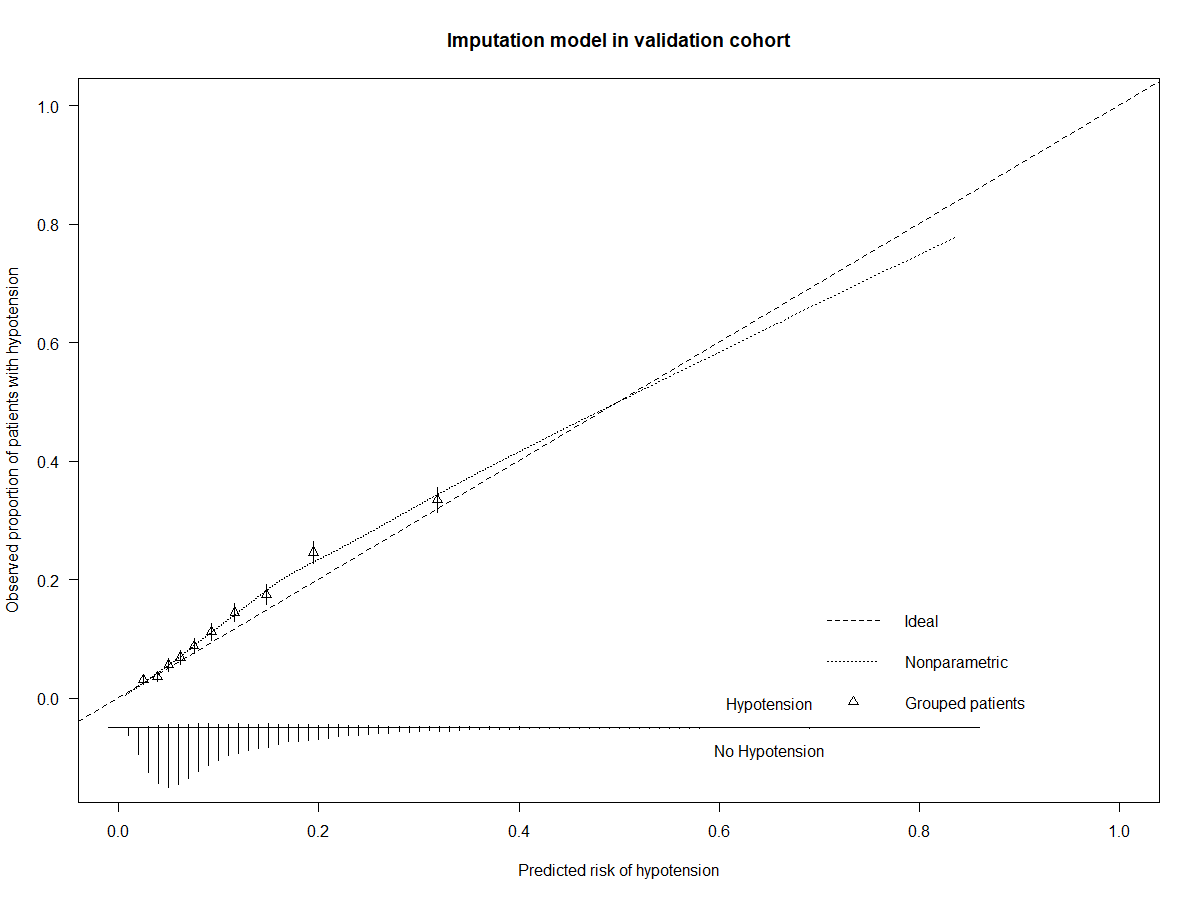


Calibration plot of the clinical prediction model using simple stochastic imputation applied to validation cohort. Y-axis: observed proportion of patients with clinically important postoperative hypotension. X-axis: predicted risk of clinically important postoperative hypotension. Ideal: line to represent perfect prediction risk equal the observed proportion of patients.

# Supplementary variable definitions

### Surgical Variables

**Major orthopaedic surgeries**: A patient undergoing one or more of the following orthopedic surgeries: major hip or pelvis surgery (hemi or total hip arthroplasty, internal fixation of hip, pelvic arthroplasty), internal fixation of femur, knee arthroplasty, above knee amputations, or lower leg amputation (amputation below knee but above foot).

**Major general surgeries:** A patient undergoing one or more of the following general surgeries: complex visceral resection (surgery involving the liver, esophagus, pancreas, or multiple organs), partial or total colectomy or stomach surgery, other intra-abdominal surgery (gallbladder, appendix, adrenals, spleen, lymph node dissection), or major head and neck resection for non-thyroid tumor.

**Major urology and gynaecology surgeries:** A patient undergoing one or more of the following major urology or gynaecology surgeries (nephrectomy, ureterectomy, bladder resection, retroperitoneal tumor resection, exenteration), cytoreduction surgery, hysterectomy, radical prostatectomy, or transurethral prostatectomy.

**Major neurosurgeries:** A patient undergoing one or more of the following neurosurgeries: craniotomy or major spine surgery (surgery involving multiple levels of the spine).

**Major vascular surgeries:** A patient undergoing one or more of the following vascular surgeries: thoracic aorta reconstructive vascular surgery, aorto-iliac reconstructive vascular surgery, peripheral vascular reconstruction without aortic cross-clamping, extracranial cerebrovascular surgery, or endovascular abdominal aortic aneurysm repair.

**Major thoracic surgeries:** A patient undergoing one or more of the following thoracic surgeries: pneumonectomy, lobectomy, other thoracic surgeries (wedge resection of lung, resection of mediastinal tumor, or major chest wall resection).

**Elective/Urgent/Emergency surgery**: Emergency surgery was surgery that occurred <24 hours after a patient developed an acute surgical condition, urgent surgery was surgery that occurred 24-72 hours after a patient developed an acute surgical condition, elective surgery was surgery that occurred >72 hours after the patient developed a surgical condition.

**Open/Endoscopic approach**: Open surgery include both open surgeries and surgeries that started endoscopically and finished open, endoscopic surgery include all endoscopic, laparoscopic, thoracoscopic, endovascular, and arthroscopic approaches.

### Patient characteristics

**Age:** The patient’s age in years, calculated as the difference between their birthdate and the date of surgery and rounded down to the nearest year.

**Preoperative hemoglobin:** Latest available routinely measured preoperative hemoglobin value.

**Preoperative estimated glomerular filtration rate (eGFR):** Calculated using CKD-Epi equation and latest available routinely measured preoperative serum creatinine value.

**Requires assistance with Activities of Daily Living**: Patient requires assistance from another person with any of the following activities: dressing, eating, ambulating, toileting, hygiene**.** If a patient has suffered an acute injury leading to the need for surgery (e.g., hip fracture) the assessment for requirement of help for ADLs was based upon their condition prior to their acute injury.

**Congestive heart failure:** A physician diagnosis of a current or prior episode of congestive heart failure or prior radiographic evidence of vascular redistribution, interstitial pulmonary edema, or frank alveolar pulmonary edema.

**Recent high-risk coronary artery disease:** Diagnosis ≤6 months prior to non-cardiac surgery of: a myocardial infarction, acute coronary syndrome, Canadian Cardiovascular Society Class (CCSC) III angina or CCSC IV angina.

CCSC III angina – angina occurring with level walking of 1-2 blocks or climbing ≤1 flight of stairs at a normal pace

CCSC IV – inability to perform any physical activity without the development of angina

**Cerebral vascular event:** A physician diagnosis of stroke, CT or MRI evidence of a prior stroke, or physician diagnosis of a prior transient ischemic attack (TIA).

**Peripheral vascular disease:** A current or prior history of: physician diagnosed intermittent claudication, vascular surgery for atherosclerotic disease, an ankle/arm systolic blood pressure ratio ≤ 0.90 in either leg at rest, or angiographic or doppler study demonstrating ≥ 70% stenosis in a non-cardiac artery.

**Chronic Obstructive Pulmonary Disease (COPD):** If the chart or a physician has ever indicated that a patient has chronic bronchitis, we accepted this as a patient having COPD. If there is no mention of this but the patient reported they have had daily production of sputum for at least 3 months in 2 consecutive years then they were marked as having COPD. Likewise, if a physician has ever indicated that a patient has emphysema or if a patient's Pulmonary Function Tests (PFT) state fixed or irreversible airflow limitation and/or emphysema then they were marked as having COPD.

**Aortic stenosis:** A physician diagnosis of aortic stenosis

**Prior cardiac arrest:** A patient with a prior history of a cardiac arrest.

**Dialysis:** defined as the use of a hemodialysis machine or peritoneal dialysis

Preoperative eGFR: Glomerular Filtration Rate estimated using the CKD-EPI equation using the most recent serum creatinine concentration measured before surgery.
